# Diffuse waxy papules and dermal mucin in skin of color: A case of scleromyxedema

**DOI:** 10.1016/j.jdcr.2025.12.005

**Published:** 2025-12-13

**Authors:** Ayushya Ajmani, Timothy Klufas, Albert Zhou, Adrienne Berke, Katalin Ferenczi, Janelle Mallett

**Affiliations:** a Geisel School of Medicine at Dartmouth, Hanover, New Hampshire; b New York Medical College, School of Medicine, Valhalla, New York; c Department of Dermatology, University of Connecticut, Farmington, Connecticut

**Keywords:** cutaneous mucinosis, dermal fibrosis, monoclonal gammopathy, mucin deposition, paraproteinemia, person of color, scleromyxedema, waxy papules

## Case report

A 62-year-old man with a history of eczema and chronic hepatitis C presented to the emergency department with facial swelling and a pruritic eruption involving the face. He reported daily cigarette smoking, alcohol consumption (40 oz beer/day), and cocaine use. Symptoms were exacerbated by food intake and persisted despite multiple courses of oral prednisone, antihistamines, and intramuscular dexamethasone. No significant improvement was noted with these therapies, and over the subsequent months, the patient developed progressive, symmetric skin thickening with waxy papules involving the glabella, scalp, face, neck, trunk, and upper extremities.

**Fig 1:**
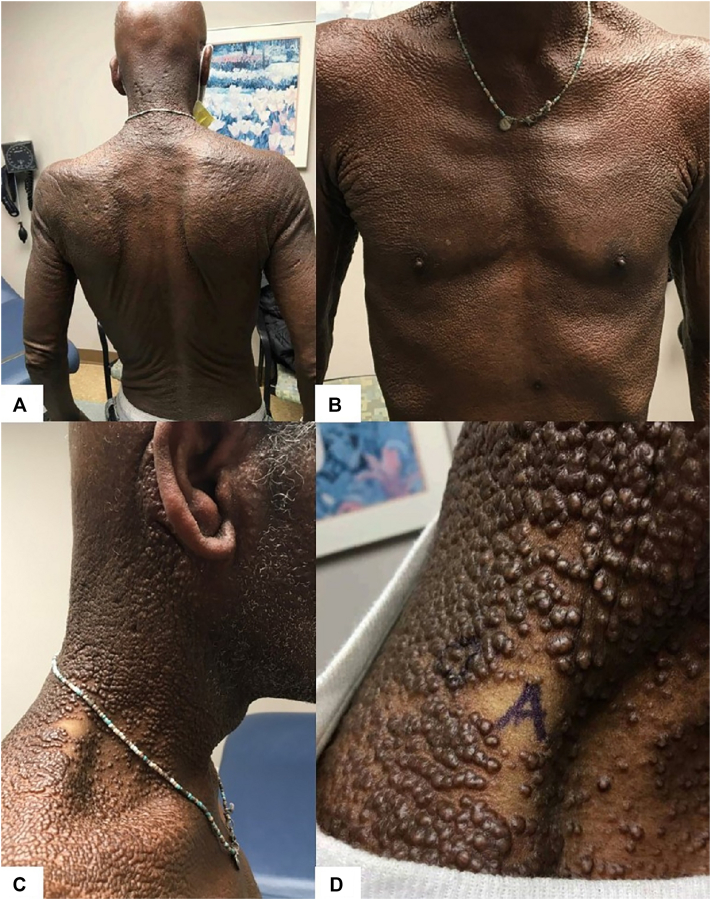
Diffuse, clustered waxy papules and indurated plaques with thickened skin on the back **(A)**, chest **(B)**, and neck **(C** and **D)**.

**Fig 2:**
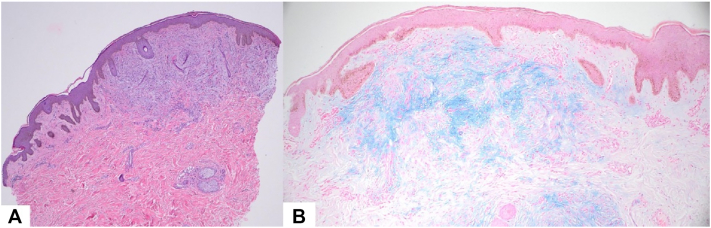
Punch biopsy showing thickened collagen bundles and increased fibroblasts on H&E **(A)**, with Alcian blue stain **(B)** highlighting diffuse dermal mucin deposition.

On examination, confluent skin-colored papules and plaques were present from the face to the upper trunk (Fig 1). Notable dermal thickening of the neck, forehead, and cheeks with mild leonine-like facies were noted. He denied weight loss, fever, or night sweats. Three 4-mm punch biopsies were obtained from the right neck, left arm, and abdomen. Histopathology revealed diffuse dermal mucin deposition (confirmed by Alcian blue staining), increased fibroblast cellularity, and thickened collagen bundles, consistent with cutaneous mucinosis (Fig 2). Direct immunofluorescence was negative.

In context of these findings, a referral to hematology was placed to rule out systemic involvement. Laboratory evaluation showed normal thyroid function and unremarkable CBC, metabolic panel, and flow cytometry. Serum protein electrophoresis revealed a restricted band (M-spike) in the gamma globulin region, and immunofixation electrophoresis confirmed a monoclonal IgG band. Findings were consistent with monoclonal gammopathy of undetermined significance without hypercalcemia, renal failure, anemia, or bone disease. Serum free light chain analysis and skeletal survey showed no evidence of multiple myeloma. Intravenous immunoglobulin (IVIG) was initiated at 2 g/kg monthly and he received 3 infusions in the following months before being lost to follow-up.

**Question: Based on the clinical presentation and histopathologic findings, what is the most likely diagnosis?**

A. Lichen myxedematosus
B. Pretibial myxedema
C. Nephrogenic systemic fibrosis
D. Scleromyxedema,
E. Alopecia mucinosa

## Discussion

The patient’s progressive, symmetric, sclerodermoid eruption with histologic findings of dermal mucin deposition, fibroblast proliferation, and collagen thickening are diagnostic of scleromyxedema (answer D). This rare, generalized form of lichen myxedematous is characterized by a triad of (1) waxy papules and skin induration, (2) mucin deposition with fibroblast proliferation on histology, and (3) an associated monoclonal gammopathy, most commonly IgG lambda, in the absence of thyroid dysfunction.^1^ Early features are often subtle and nonspecific, leading to misdiagnoses like atopic dermatitis or keratosis pilaris. Disease progression typically involves the face, neck, and upper trunk, and may affect neurologic, pulmonary, gastrointestinal, and rheumatologic systems.^1^

The pathogenesis is poorly understood, however, it is hypothesized profibrotic cytokines such as IL-1, TNF-α, TGF-β, and IL-4, stimulate fibroblast activity and glycosaminoglycan deposition.^2^ A Th2-skewed immune response with reduced interferon-γ and IL-17 production further supports this fibroinflammatory cascade.^3^ Although paraproteins are nearly always present, their direct pathogenicity remains debated, and they do not correlate reliably with disease severity.

First-line therapy is IVIG, typically administered at 2 g/kg monthly. IVIG improves cutaneous and systemic symptoms and is well-tolerated.^4^ Combination approaches using immunomodulators or proteasome inhibitors such as bortezomib may be considered in refractory cases.^5^ Corticosteroids and antihistamines are typically insufficient as monotherapy. Scleromyxedema may necessitate interdisciplinary care, given the 10% chance of progression into multiple myeloma.

## Conflicts of interest

None disclosed.
